# Use of drugs, perceived drug efficacy and preferred providers for febrile children: implications for home management of fever

**DOI:** 10.1186/1475-2875-8-131

**Published:** 2009-06-12

**Authors:** Elizeus Rutebemberwa, Xavier Nsabagasani, George Pariyo, Goran Tomson, Stefan Peterson, Karin Kallander

**Affiliations:** 1 Department of Health Policy, Planning and Management, Makerere University School of Public Health, PO Box 7072, Kampala, Uganda; 2 Division of Global Health, IHCAR, Department of Public Health Sciences, Karolinska Institutet, SE 17177 Stockholm, Sweden; 3 Northern Uganda Transition Initiative, Plot No 1, Samuel Doe Road, PO Box 1521, Gulu, Uganda; 4 Medical Management Centre, Karolinska Institutet, Stockholm, Sweden; 5 International Maternal and Child Health Unit, Department of Women's and Children's Health, Uppsala University, Sweden; 6 Department of Epidemiology and Biostatistics, Makerere University School of Public Health, PO Box 7072, Kampala, Uganda

## Abstract

**Background:**

Community distribution of anti-malarials and antibiotics has been recommended as a strategy to reduce the under-five mortality due to febrile illnesses in sub-Saharan Africa. However, drugs distributed in these interventions have been considered weak by some caretakers and utilization of community medicine distributors has been low. The aim of the study was to explore caretakers' use of drugs, perceptions of drug efficacy and preferred providers for febrile children in order to make suggestions for community management of pneumonia and malaria.

**Methods:**

The study was conducted in eastern Uganda using four focus group discussions with fathers and mothers of children under five; and eight key informant interviews with health workers in government and non-governmental organization facilities, community medicine distributors, and attendants in drug shops and private clinics. Caretakers were asked the drugs they use for treatment of fever, why they considered them efficacious, and the providers they go to and why they go there. Health providers were interviewed on their opinions of caretakers' perceptions of drugs and providers. Analysis was done using content analysis.

**Results:**

Drugs that have been phased out as first-line treatment for malaria, such as chloroquine and sulphadoxine/pyrimethamine, are still perceived as efficacious. Use of drugs depended on perception of the disease, cost and drug availability. There were divergent views about drug efficacy concerning drug combinations, side effects, packaging, or using drugs over time. Bitter taste and high cost signified high efficacy for anti-malarials. Government facilities were preferred for conducting diagnostic investigations and attending to serious illnesses, but often lacked drugs and did not treat people fast. Drug shops were preferred for having a variety of drugs, attending to clients promptly and offering treatment on credit. However, drug shops were considered disadvantageous since they lacked diagnostic capability and had unqualified providers.

**Conclusion:**

Community views about drug efficacy are divergent and some may divert caretakers from obtaining efficacious drugs for febrile illness. Interventions should address these perceptions, equip community medicine distributors with capacity to do diagnostic investigations and provide a constant supply of drugs. Subsidized efficacious drugs could be made available in the private sector.

## Background

Febrile illnesses like malaria and pneumonia are major contributors to the high child mortality rates in sub-Saharan Africa [1]. Various countries committed themselves to reduce child mortality rates by two thirds by 2015 [2]. The distribution of anti-malarials and antibiotics at community level by community medicine distributors (CMDs) [3,4] is one of the interventions recommended to reduce mortality from febrile illnesses. This strategy has been shown to reduce morbidity in Burkina Faso [5] under five mortality due to malaria in Ethiopia [6] and mortality due to pneumonia in Nepal [7]. A meta-analysis of community case management of pneumonia in India, Pakistan, Bangladesh, Philippines and Tanzania showed a mortality reduction of 27% [8,9]. The momentum of introducing community case management of pneumonia is high in Africa [10] and national programmes for community health workers are being planned in Ethiopia, India, Kenya, Uganda and South Africa [11].

However, drugs distributed by CMDs have been perceived by some caretakers as weak [12] or ineffective [13]. Community management of febrile illnesses has used pre-packed drugs [14], but some women in western Uganda did not like pre-packed drugs [15]. New drugs are to be distributed, which are different from the previous ones. Caretakers trusted in the efficacy of chloroquine as an anti-malarial due to its bitter taste [16]. Artemether/lumefantrine (AL), the new first-line treatment of malaria is not bitter. Cotrimoxazole is the first-line of treatment for pneumonia in Uganda, but a high in vitro resistance has been reported [17]. Amoxicillin is the second drug of choice. Studies differ on caretaker use of antibiotics with some showing high utilization [18] and others showing that antibiotics are not cited in the treatment for fever [19]. Much of the community management of pneumonia has been in Asia and few studies have taken place in sub-Saharan Africa. Even in the presence of CMDs, some caretakers prefer to go to drug shops and private clinics [12]. Utilization of CMDs was noted to be low where the community distribution of anti-malarials was done in Democratic Republic of Congo [20], The Gambia [21], Uganda [14] and Kenya [22].

If the community distribution of anti-malarials and antibiotics is to have an impact on child mortality, it needs to be used by a big proportion of the febrile children. The strategy needs to offer drugs that are seen by caretakers as efficacious. The CMDs need to be seen as providers that would manage malaria and pneumonia. The aim of the study was to explore caretakers' use of drugs, perceptions of drug efficacy and preferred providers and make suggestions for the distribution of anti-malarials and antibiotics at community level.

## Methods

### Study area

The study was done in Iganga district in eastern Uganda, located about 115 km from the capital Kampala. The district population is about 661,400 (2008 estimate). Malaria is endemic in the area and the under five mortality rate for the region is 128/1,000 live births [23]. The majority of the population are Basoga who speak Lusoga and are mostly engaged in subsistence farming. The area is served by 58 government and 23 non-governmental organization (NGO) health facilities. Many of the government facilities frequently lack drugs. There are 110 registered drug shops and 36 registered private clinics, especially in trading centres, which sell analgesics, such as paracetamol (Panadol^®^) and aspirin, anti-malarials, such as chloroquine, sulphadoxine/pyrimethamine (SP)(Fansidar^®^) and quinine, and antibiotics, such as cotrimoxazole and amoxicillin. Like in many other parts of the country, many of the drug shops and private clinics are not registered [24,25]. Artemether/lumefantrine (AL)(Coartem^®^) is currently distributed through government and NGO health facilities and its distribution by CMDs has not yet been implemented [26]. There was no community distribution of anti-malarials and antibiotics at the time of the study. However, before the change of first-line anti-malarials from chloroquine and SP to AL, there were 960 trained and functional CMDs in the district. They used to distribute chloroquine and SP free-of-charge to under five children at community level. Since AL is very expensive on the open market, most drug shops and private clinics in the rural areas did not stock it [27]. There were also traditional healers scattered in the villages.

### Study population and data collection techniques

The data collection methods included focus group discussions (FGDs) and key informant interviews (KIIs). Four FGDs were conducted: two with fathers and another two with mothers. Each FGD was conducted in a different sub-county. Two of the FGDs were in sub-counties close to and another two in sub-counties far away from the district headquarters. Whereas most fevers are managed at clinics and drug shops by mothers, FGDs of fathers were conducted because fathers also play a significant role in the health care seeking of febrile children, especially when costs are to be incurred. All FGD participants had children below five years and were known residents of the area. They were mobilized by a community member identified by the research group. They were considered 'information rich cases' [28], as they were in a good position to discuss experiences about childhood illnesses. Each FGD had between nine and 12 participants. FGDs were conducted in one of the homes of the FGD participants.

FGDs were used to generate debate and allow the participants explore their views and opinions about drugs and their efficacy and preferences for providers [29]. By having fathers and mothers alone, the FGDs gave a chance to mothers, who may not have expressed themselves freely in the presence of men, to do so. FGDs give an opportunity for the researchers to listen to people who have little chance of expressing their opinions [30]. Both the interviewer and the note taker were social scientists and experienced in conducting FGDs. They spoke both English and the local language fluently. The first FGD, which was with women, was attended by ER and XN. Afterwards, ER, XN and KK went through the notes. They discussed with the interviewer and the note taker their experiences of the emerging issues and whether questions were being understood. An efficacious drug was described as one which was powerful hence able to cure the disease and a non-efficacious drug as that which was weak and would not be able to cure the disease when used. The FGDs were conducted using an FGD guide which focused on drugs that caretakers give children with fever or fever and cough, the drugs they consider efficacious, those they consider non-efficacious and why. Other questions focussed on which providers caretakers go to when the children present with fever and why they go to such providers.

Eight KIIs were conducted by an experienced social worker fluent in both English and the local language. Two of the KIIs were held with health workers from two of the local health facilities (one being government and another NGO), two with attendants in drug shops, two with attendants in private clinics and two with people who had been functioning as community medicine distributors before. The six respondents from the drug shops, private clinics and public health facilities were: two clinical officers, two nurses, one mid wife and one nursing assistant. Each came from a different health facility, drug shop or clinic. Those interviewed as former CMDs were the community members who had functioned as CMDs, when chloroquine and SP were still being distributed free at community level. By the time of the interview, they were not actively distributing any drug within the community. Respondents in KIIs were chosen because they would have interacted closely with caretakers of febrile children. Key informant interviews were conducted to capture the experiences of the providers as they do interface with the caretakers when the latter come for health care. Four KI interviews were conducted first and then data reviewed by ER and XN to assess how the questions were being answered before the other four interviews were done. The guiding questions for the KIIs included identification of the drugs, which the caretakers used for malaria and for pneumonia, which of the drugs caretakers considered efficacious and why. Other questions focussed on health providers to whom caretakers went to when their children were febrile and why they preferred such providers.

The research assistants were trained by the investigators on how to use the tools. The data was collected in July – August 2008. All the data were tape recorded and transcribed. The FGDs were conducted in the local language, transcribed and later translated into English by the interviewers. The researchers listened to the tapes to confirm the information.

### Data analysis

Content analysis was used [31]. The unit of analysis was the transcripts from FGDs and KIIs. The authors read through the data and discussed, sometimes coming up with different issues and debating on them and eventually came up with codes. After the first discussion, ER, XN and KK went back and read through the material again after which they met and generated more codes, which were discussed and agreed upon. These codes were merged into categories and then into themes.

### Ethical clearance

The study was approved by Makerere University School of Public Health Institutional Review Board and the Uganda National Council of Science and Technology (HS 72). Permission to carry out the study was received from the local leaders. Verbal informed consent was received from all the participants.

## Results

There were four key findings of the study: (1) Quite often respondents were not up-to-date with the newly recommended drugs as some of them said they were still using chloroquine and/or SP for fever. They also tended to mix anti-malarials and antibiotics as belonging to one category. Drugs used for fever that were commonly mentioned included analgesics, anti-malarials, antibiotics, anticonvulsants, steroids and traditional medicines. (2) Caretakers gave divergent views about efficacy of different drug combinations, packaged drugs and significance of side effects. (3) Both private and public health providers were used for treating febrile illness, each in specific circumstances. (4) The ideal provider was that who had diagnostic capability, was nearby, available all the time and provided a constant supply of a variety of drugs.

### Caretakers' use of drugs

Drugs used by caretakers to treat febrile children included analgesics, such as Panadol^®^, diclofenac^® ^and ibuprofen; anti-malarials, such as chloroquine, Fansidar^®^, quinine and Coartem^®^. Other drugs included diazepam, dexamethasone and traditional herbs like "lubirizi"*(Vernonia amygdalina) *and "akabombo akaganda" (*Cyphostemma adenocaule)*. Chloroquine and Panadol^® ^were the commonest drugs cited for treating fever. For treating cough, the majority mentioned septrin (cotrimoxazole). Other drugs included chloramphenicol syrup, ampicillin syrup, PPF (procaine penicillin) and herbs, like aloevera *(Aloe vera chinensis) *and lemon leaves. Majority of the participants indicated that they would give a sick child drugs they would either already have at home or getting them from a drug shop. Care from health facilities would be sought when the child would not improve. Traditional medicine for treating fever was known by the caretakers themselves and they did not need to get it from a traditional healer.

Some of the drugs considered weak were used as first aid and more powerful ones as last resort. Drugs perceived to be weak included chloroquine, Fansidar^®^, Panadol^®^, diclofenac and septrin. However, there were some of the respondents who perceived these drugs to be efficacious and were using them to treat children. According to the majority of the caretakers, even weak drugs had an important role to play.

*For me what I know is that there is no treatment, which does not work because it only depends on the severity of the fever*. (FGD women)

Quinine was considered powerful and often used as last resort.

*For us we know that we first begin with a weak drug and advance to powerful drugs as the sickness worsens. Quinine is given last when the other drugs have failed*. (FGD Men)

This was in agreement with opinions of providers on caretakers:

*Parents consider chloroquine a weak drug but they also think that if the child has slight malaria, chloroquine can be adequate and can help the person to get cured*. (KI former CMD)

Use of drugs was also influenced by how much money one could spend and presence of the drug on the market.

*Capsules are very expensive so I rather settle for the tablets like septrin because one capsule costs 100/= and for 100/= you get 8 septrin tablets." *(FGD women)

*Sometimes money is also a determinant. The child can be four months and you advise the caretaker to use quinine syrup but the caretaker will tell you that she has very little money so you give them chloroquine tablets*. (KI Drug shop attendant)

*Most caretakers say septrin is weak yet we have it on open market.... we have to buy septrin tablets and give them to the patients because they are on open market*. (KI clinic attendant)

### Caretakers' perception of drug efficacy

Views on drug efficacy were diverse and there were often disagreements on which drug is efficacious when combined with others, what side effects show about drug efficacy, packaging, what prompt recovery shows about the drug taken and efficacy of using a drug for some time. Taste and cost as indicators of efficacy were discussed though without much disagreement.

The majority of the participants argued that drugs are effective when given in combinations. They maintained that there are some drugs which cannot work unless they are combined with others. Some participants thought Panadol^® ^was best because it is given with other drugs, like Fansidar^®^. Some other participants perceived a drug like Panadol^®^, which is usually added onto other drugs, as weak.

There were many considerations that made people conclude that a drug was efficacious. Such considerations included side effects, packaging, the drug source, how quickly the body responded to the drug. There was no consensus on the implications of a drug having side effects. For some, it was a sign of a drug being strong.

*For me, the drug that cures very well is quinine. Even when you take quinine you can feel it. It is very strong because you even feel pain in the ears after taking it*. (FGD Men)

*Sometimes if they give a drug to a child and he/she weakens then they say the drug is strong*. (KI drug shop attendant)

While others argued that when a drug has no side effects, it is a sign that the drug is efficacious and appropriate for the disease in question.

*Once the drug is given to the child and does not involve any complications like convulsions, vomiting or shivering, then I know that the drug is recommended for malaria and I know that that drug works*. (FGD Men)

There were also diverse views on packaging with some saying that pre-packaged drugs were the ones which were efficacious.

*Those drugs that are not in the blister pack are the ones that are most likely to be duplicated. Me I prefer the ones in the blister seal*. (FGD Men)

However, there were others who on the contrary argued that since the government health facilities usually had the tablets that are not packed; those in blister packs were fake.

*I think that the drugs in the blister seal are not as original as the other common ones (loose ones) because even government has the loose ones which they keep in the tins, so I think that the ones in the tins are the original ones and those in blister packs are fake*.(FGD men).

Drugs that had immediate effect on the condition of the child were considered by some of the respondents as efficacious. Other participants argued to the contrary and said that no drug cures immediately.

*There is no medicine that cures immediately, they all work gradually because diseases come quickly but go slowly*. (FGD women)

There were disagreements as to what happens when a person has used a drug for some time. Some held the view that when a person uses a drug for some time, the drug would no longer cure that particular person.

*On my side, quinine works well on my child but these days when I use it, the fever resumes after some time. ... May be the child's body has gotten used to quinine and the child's body is not responding hence quinine cannot treat the fever any more*. (FGD women)

Experience with the use of some of the drugs influenced the way people perceived the drugs.. It was common for people to refer to the drugs they use often as being less efficacious. Other participants had dissenting views suggesting that people are cured by certain drugs which they have gotten used to.

*Some people use chloroquine injections but they fail to get cured from the fever because they are used to another type of treatment. For instance I may use chloroquine injections and I do not get cured and yet when I use fansidar tablets, I get cured*. (FGD women)

A summary of divergences in perceptions of drug efficacy is shown in Table 1.

**Table 1:** Divergences in perceptions of drug efficacy

**Description**	**One opinion**	**Another divergent opinion**
1. When using drugs in combination	A powerful drug is found in drug combinations	A weak drug is the one in drug combinations

2. Drugs having side effects	This is a sign that a drug is efficacious	This is a sign that a drug is not efficacious

3. Pre-packaged drugs	Are the ones which are efficacious	Are not efficacious, they are fake

4. Drugs giving fast recovery from the illness	Shows that the drug is efficacious	There is no drug that gives quick recovery. All drugs act slowly

5. Effect of using a drug for some time	Makes the person be cured by preferably that drug	Makes a person unable to be cured by that drug

Most of the participants were of the opinion that having a bitter taste was a sign of a drug being efficacious for the treatment of malaria. In addition, a big section of the FGD participants held the view that expensive drugs were more efficacious than the cheap ones.

*I think that all drugs that cure malaria should be bitter... this is because malaria is strong and therefore needs some mixture that is equally bitter. Me, if I tested a medicine for malaria that's not bitter, I would know that it is just a fake one and I would not accept it, I can't purchase it*. (FGD Men)

*For us here, if there is any medicine that's cheap, we suspect that where as it is used to treat malaria, it may not work well. We believe that certain drugs are expensive because they work. For example when you buy 10 chloroquine tablets, a dose may be only 200/= while a dose of only 3 fansidar tablets is at 1500/=. So fansidar is seen as being more powerful *(FGD Men)

### Preference for providers

Both private and public providers were used for managing febrile illness. However, the preferences were for providers: where there was no waiting, open all the time, which were nearby, could give treatment on credit, had drugs, and had diagnostic capability.

The majority of the participants credited drug shops for being able to give treatment promptly unlike government facilities, which do not even give special attention to very sick children.

*Unlike government facilities, private clinics and drug shops care about their clients. Once you reach there, you are welcomed nicely and served immediately, but at the government health facility you can enter at 9:00 am and the doctor may or may not attend to you at 1:00 pm, so in cases where you need immediate attention you have no option but to go to the private clinic or drug shop*.(FGD Men)

*When you go to the main hospital you make a line even when the child is in poor condition and the child may die before being attended to*. (FGD women)

Drug shops were also commended by most of the respondents for being near and being able to give services even at night unlike the government facilities that were far.

*For us when get sick from malaria we get treatment through buying these drugs from the drug shops because even our government facility is very far away from here. For example a child can fall sick at night so I have to get the treatment from the drug shop other than go at night to the government facility*. (FGD women)

*The drug shops have a range of drugs on the shelves and can give you the quantities you want, they can mix for you the drugs according to your money, they are also near us and since we are the same customers, they can give you treatment on credit and may even bargain for a cheaper cost*. (FGD Men)

*When you go to Nakavule (the general hospital in the district), you need to use a motor cycle yet if you have 500/= you can go to the nearby drug shop and get drugs *(KI former CMD)

However, drug shops and private clinics had drawbacks. Few of the FGD participants said that these private providers sometimes used nursing assistants without enough qualifications. The caretakers would still to go to them because of having no other choice. Drug shops did not do laboratory investigations and hence were perceived to treat the children basing on symptoms. To some participants, this was tantamount to guesswork.

*I can get medicine like chloroquine from the drug shops although the nursing assistant who is there does not have enough qualifications but I have no option but to go to them*. (FGD Women)

*Now here we have a problem when a child is sick, the drug shop attendants just start on treatment without checking the child's blood to see what is in the blood to see the disease. When you take the child to the drug shop, they just put it on treatment*. (FGD women)

Some caretakers were of the opinion that government facilities had the new and efficacious drug against malaria (Coartem^®^). When children needed intravenous fluids or blood, they had to be taken to the government hospital. They also had capacity to do laboratory investigations. This was also reflected in a comment from a former CMD who praised services at government facilities because there was proper diagnostic equipment;

*Government facilities are good because if one falls sick they are advised to go to a health facility so that they can have a blood checkup. When my child fell sick, I was advised to take the child to the hospital for a blood test so that she can receive the appropriate treatment*. (KI former CMD)

Table 2 summarizes preferred provider attributes.

**Table 2:** Preferred provider attributes

**Attribute**	**Government facility**	**Private clinic or drug shop**	**Comments**
1. Attend to clients fast	No	Yes	Some times one waits at government facilities and does not get treatment

2. Can attend to the child any time of day or night	No	Yes	At night it is difficult to go to government facilities because they are far

3. Are nearby	No	Yes	Sometimes the cost of transport is more than the cost of treatment

4. Have drugs in constant supply	No	Yes	Drug shops do not have the new anti-malarials of Coartem^®^

5. Can conduct investigations	Yes	No	Investigations are needed to find out why previous treatment did not work

6. Have qualified workers	Yes	No	Drug shops have unqualified workers but there is no choice, caretakers have to use them

7. Handle complicated illnesses	Yes	No	Without the lab, there is need for investigations and drug shops do not have that capacity

## Discussion

Drugs which caretakers consider 'weak' are still being used sometimes as a form of 'first aid' and sometimes as treatment. Caretakers' perceptions on drug efficacy are not consistent and include diverse considerations on efficacy of drugs used in combinations, significance of side effects, efficacy of pre-packed drugs and significance of prompt recovery. Caretakers' ideal providers are those who offer investigations and have a variety of drugs. Whereas government facilities are preferred for being able to conduct diagnostic investigations and handling serious illnesses, they are often short of drugs. Drug shops are the ones that can supply constantly a variety of drugs, offer treatment promptly and have convenient opening hours.

Caretakers more often use weak drugs in treating febrile children. Chloroquine and paracetamol are the commonest treatment for fever. There is much resistance to chloroquine and SP [32]. This means that people use drugs which are less efficacious. As efficacy of a drug declines, children would not improve on drugs that they previously improved on. Drugs that were distributed by CMDs at first were chloroquine and SP [12] but with time, their efficacy went down. This sheds more light on why drugs from CMDs were considered weak [12-14]. However, caretakers still use these drugs. The Uganda Demographic and Health survey recorded a high utilization of chloroquine and SP for treating febrile children [33]. Non-efficacious drugs could be used because they are cheap or because the efficacious ones are not in the drug shops in the rural areas [27]. Previous studies have demonstrated that it is feasible to distribute efficacious drugs with CMDs [34] and drug shops [35]. Although drug shops have been associated with promptness of treatment for febrile children [36]they are not providing the recommended drugs as per the new treatment guidelines. This continued use of non-efficacious anti-malarials brings challenges to effective treatment of malaria.

The study findings indicate that the perceptions about drugs, which are efficacious cannot be generalized. There were differences in opinion about drugs, which come in combinations, the significance of side effects, drug packaging and implications of time of symptom clearance. Most caretakers agreed that drug combinations are efficacious. This could have been a product of policy by combining chloroquine and SP or artemether and lumefantrine. There has also been occasions of poly pharmacy in drug utilization in Africa just because of the "pill for every ill" [37,38]. In the "pill for every ill" concept, a patient with multiple symptoms like hot body, headache, dizziness and joint pains, all of which may be symptoms of malaria, could be treated with one pill for hot body, another for headache, another for dizziness and even with another for joint pains instead of the one pill anti-malarial. Other studies have reported that CMDs were not utilized because of lack of the combination aspect-they only provided one drug [12]. Not many studies have highlighted side effects as influencing perceptions about drug efficacy. Despite previous history of community management of fever [14], there are still disagreements on efficacy of packaged drugs. Seeking quick recovery could partly explain the popularity of analgesics and chloroquine, which also has antipyretic properties. Based on these findings it is still difficult to point out the drugs that are perceived to be efficacious generally. It is important to note that whenever a drug works for caretakers, the drug is efficacious and there is general tendency to think that some drugs work for some children and not others [12]. Some of these perceptions like associating efficacy with drugs that are not pre-packaged may divert caretakers from obtaining pre-packaged drugs from CMDs and need to be addressed in interventions.

In this study, a common belief was that people may get used to a drug, which means that the drug cannot be used to cure them next time. Conversely, other participants argued that only drugs that the body has gotten used to can cure the illness. This could be a community way of explaining drug failure, stemming from the common caretaker use of drugs with reduced efficacy in treating malaria. There has been an increase in malaria parasite resistance to chloroquine and SP [39,40]. The first-line treatment for malaria has been changed to AL. Different scenarios could explain this drug failure. It is possible that caretakers use drugs like chloroquine and SP that have reduced efficacy because they are still on the open market. It is also possible that the caretakers give a wrong medicine like an anti-malarial drug to a child with pneumonia and say that the drug is less efficacious [19]. The *emic *concepts – cultural constructs of efficacy – often differ from the *etic *explanations – concepts with scientific explanation [41]. While biomedical efficacy would be tagged to clearance of malarial parasites in blood, caretakers would be satisfied with lowering of body temperature or as indicated by some, presence of side effects like noise in the ears after taking quinine. Another possibility could be that the caretakers have not yet adopted the new first line drug for malaria treatment. Communities have been shown to be slow to take on new policies as demonstrated in Tanzania [42]. It becomes more problematic when the drug shops where the caretakers seek treatment first do not have first line treatment for malaria [27]. Drug shops provide drugs in respect for consumer preferences and the client's ability to pay for the drugs. They often do not consider the symptoms but rather what the person requests. Because people do not have a clear understanding of the right drugs for fever, they often ask for wrong drugs and sometimes take under dose, which they can afford. Efficacious drugs remain outside the reach of the majority when drugs are difficult to obtain from outside the government health facilities [43].

Community interventions could build on the preference of caretakers for drug shops because the latter offered a constant supply of drugs, treated clients promptly, gave treatment on credit, and were nearby. This has influenced caretakers to choose them for care [44,45]. Some of the challenges levelled against the home based management of fevers have been frequent stock outs of drugs at the CMDs [46]. Frequent stock outs will drive caretakers to drug shops who don't stock the first-line anti-malarials (artemether/lumefantrine) [27].

Caretakers prefer government facilities when children have more serious symptoms because the government facilities are able to conduct laboratory investigations. When caretakers take children for treatment outside the home, they want to know the cause of the illness that is not responding to treatment. The acceptance of CMDs could potentially increase if they were equipped with diagnostic tests such as rapid diagnostic tests (RDTs) for malaria. RDTs have been promoted in situations where there are no laboratories for microscopy [47] and are being used to identify cases of malaria at community level so as not to give artemether/lumefantrine, an expensive anti-malarial, to any child with fever [48]. Drug shops have been shown to offer poor quality [25,49]. However, febrile children would benefit if multifaceted interventions including training, supervision, and regulation were extended to drug shops and private clinics [50].

### Methodological considerations

Using different methods and respondents, and having researchers from different backgrounds from social science, medicine and the basic sciences was aimed at getting an objective perspective of the data. The study was faced with the challenge of the limitations of the qualitative methods, which do not give the magnitude across the different categories of the respondents. However triangulation of information across caretakers and health providers, across mothers and fathers, using both FGDs and KIIs, was very useful in checking consistency and contradictions both across and within groups [51,52]. The use of purposively selected participants makes the research findings not generalizable to the general population. Another limitation is that the study reports the caretakers' *stated *use of drugs rather than *actual *practices. However, separating women from men in the FGDs and asking questions focussed on common health care-seeking practices could have promoted free expression of the participants and also reduced answers given to please the researcher. Further studies will be needed to address the impact of these caretakers' perceptions on the actual care that the febrile children receive.

### Conclusion and recommendations

Caretakers sometimes use what they perceive as 'weak' drugs as 'first aid' and if the child does not get well, go for more powerful drugs. This aggravates the problem of drug resistance and misuse. There are divergent views among caretakers on what factors imply that a drug is efficacious. Some perceptions may divert caretakers from using pre-packaged drugs from CMDs. As caretakers would prefer to get the drugs from providers that have diagnostic ability, attend to clients fast, and are available all the time, nearby, trained and have a constant supply of efficacious drugs, there is great potential for community distribution of anti-malarials and antibiotics to meet community expectations if CMDs live up to these standards. To increase the success of the home management of fever strategy, there is need to develop strategies for constant drug supply, to sensitize caretakers on which drugs are currently efficacious, and to avail subsidized efficacious drugs also in the private sector.

## Competing interests

The authors declare that they have no competing interests.

## Authors' contributions

ER, XN, GP, GT, SP and KK took part in designing the study, in tools development, in data analysis and in manuscript writing. ER, XN and KK did field work. All authors approved the final manuscript.
